# PrEvention of posttraumatic contractuRes with Ketotifen 2 (PERK 2) – protocol for a multicenter randomized clinical trial

**DOI:** 10.1186/s12891-020-3139-2

**Published:** 2020-02-24

**Authors:** Ayoola Ademola, Kevin A. Hildebrand, Prism S. Schneider, Nicholas G. H. Mohtadi, Neil J. White, Michael J. Bosse, Alexandra Garven, Richard E. A. Walker, Tolulope T. Sajobi

**Affiliations:** 1 McCaig Institute of Bone and Joint, University of Calgary, Calgary, Canada; 2 Department of Community Health Sciences, University of Calgary, Calgary, Canada; 3 Department of Surgery, University of Calgary, Calgary, Canada; 4 Department of Orthopaedic Surgery, Carolinas Medical Center, Charlotte, USA; 5 Department of Radiology, University of Calgary, Calgary, Canada

**Keywords:** RCT, Ketotifen Fumarate, Contractures, DASH, OES, PCS, ROM

## Abstract

**Background:**

Injuries and resulting stiffness around joints, especially the elbow, have huge psychological effects by reducing quality of life through interference with normal daily activities such as feeding, dressing, grooming, and reaching for objects. Over the last several years and through numerous research results, the myofibroblast-mast cell-neuropeptide axis of fibrosis had been implicated in post-traumatic joint contractures. Pre-clinical models and a pilot randomized clinical trial (RCT) demonstrated the feasibility and safety of using Ketotifen Fumarate (KF), a mast cell stabilizer to prevent elbow joint contractures. This study aims to evaluate the efficacy of KF in reducing joint contracture severity in adult participants with operately treated elbow fractures and/or dislocations.

**Methods/design:**

A Phase III randomized, controlled, double-blinded multicentre trial with 3 parallel groups (KF 2 mg or 5 mg or lactose placebo twice daily orally for 6 weeks). The study population consist of adults who are at least 18 years old and within 7 days of injury. The types of injuries are distal humerus (AO/OTA type 13) and/or proximal ulna and/or proximal radius fractures (AO/OTA type 2 U1 and/or 2R1) and/or elbow dislocations (open fractures with or without nerve injury may be included). A stratified randomization scheme by hospital site will be used to assign eligible participants to the groups in a 1:1:1 ratio. The primary outcome is change in elbow flexion-extension range of motion (ROM) arc from baseline to 12 weeks post-randomization. The secondary outcomes are changes in ROM from baseline to 6, 24 & 52 weeks, PROMs at 2, 6, 12, 24 & 52 weeks and impact of KF on safety including serious adverse events and fracture healing. Descriptive analysis for all outcomes will be reported and ANCOVA be used to evaluate the efficacy KF over lactose placebo with respect to the improvement in ROM.

**Discussion:**

The results of this study will provide evidence for the use of KF in reducing post-traumatic joint contractures and improving quality of life after joint injuries.

**Trial registration:**

This study was prospectively registered (July 10, 2018) with ClinicalTrials.gov reference: NCT03582176.

## Background

Joint contractures develop as a consequence of trauma, arthritis, or reconstructive procedures and can be functionally debilitating [1]. These injuries occur most commonly in the working age group (20–60 years), thus, representing a significant societal burden in North America [2–5]. Analysis of the Calgary Health Region database revealed that approximately 1200 elbow fractures or dislocations occurred in 2002–2005 [2–5]. These rates extrapolated to the Canadian population resulted in an estimated 20,000 elbow fractures or dislocations per year nationally. Assuming 1 in 8 will develop a joint contracture requiring operative intervention, an estimated > 2500 operative procedures per year for elbow contracture in Canada [6–10]. Extrapolated to the US, this would yield over 25,000 operative procedures annually.

Over the last 20 years, our laboratory research has implicated a myofibroblast-mast cell-neuropeptide axis of fibrosis in post-traumatic joint contractures [11]. Ketotifen Fumarate (KF) is a medication that has anti-anaphylactic properties, due to the prevention of the synthesis and/or release of growth factors and mediators from mast cells. It has been used in the treatment of chronic asthma for over 40 years in humans. Post-market surveillance has confirmed the safety of KF (11, Bassler). Through a preclinical rabbit model of post-traumatic joint contractures, we have shown that KF, decreased contracture severity concomitant with decreased numbers of myofibroblasts, mast cells, neuropeptide containing nerve fibres, and measures of fibrosis in the joint capsule in a dose-dependent fashion [12–14]. It is the first and only agent demonstrating both a significant decrease in contracture severity in preclinical trials and a wide safety profile. A previously conducted pilot randomized clinical trial (NCT01902017) demonstrated safety of KF and coupled with preclinical animal studies informed the need to increase the sample size, use multiple doses to examine for a dose response, and further refine the trial population to more severe injuries requiring an operation in the Phase III randomized clinical trial (RCT).

The primary objective is to evaluate the efficacy of KF in reducing post-traumatic elbow joint contractures when compared to placebo in participants with elbow fractures or dislocations administered within 7 days of injury and for a duration of 6 weeks. There are two secondary objectives i.e. (1) Evaluate the efficacy of KF over lactose placebo with respect to Disability Arm Shoulder Hand (DASH), Oxford Elbow Score (OES), and Pain Catastrophizing Scale (PCS), (2) Evaluate the impact of KF on safety including serious adverse events, fracture healing and re-operation rates.

**Fig. 1:**
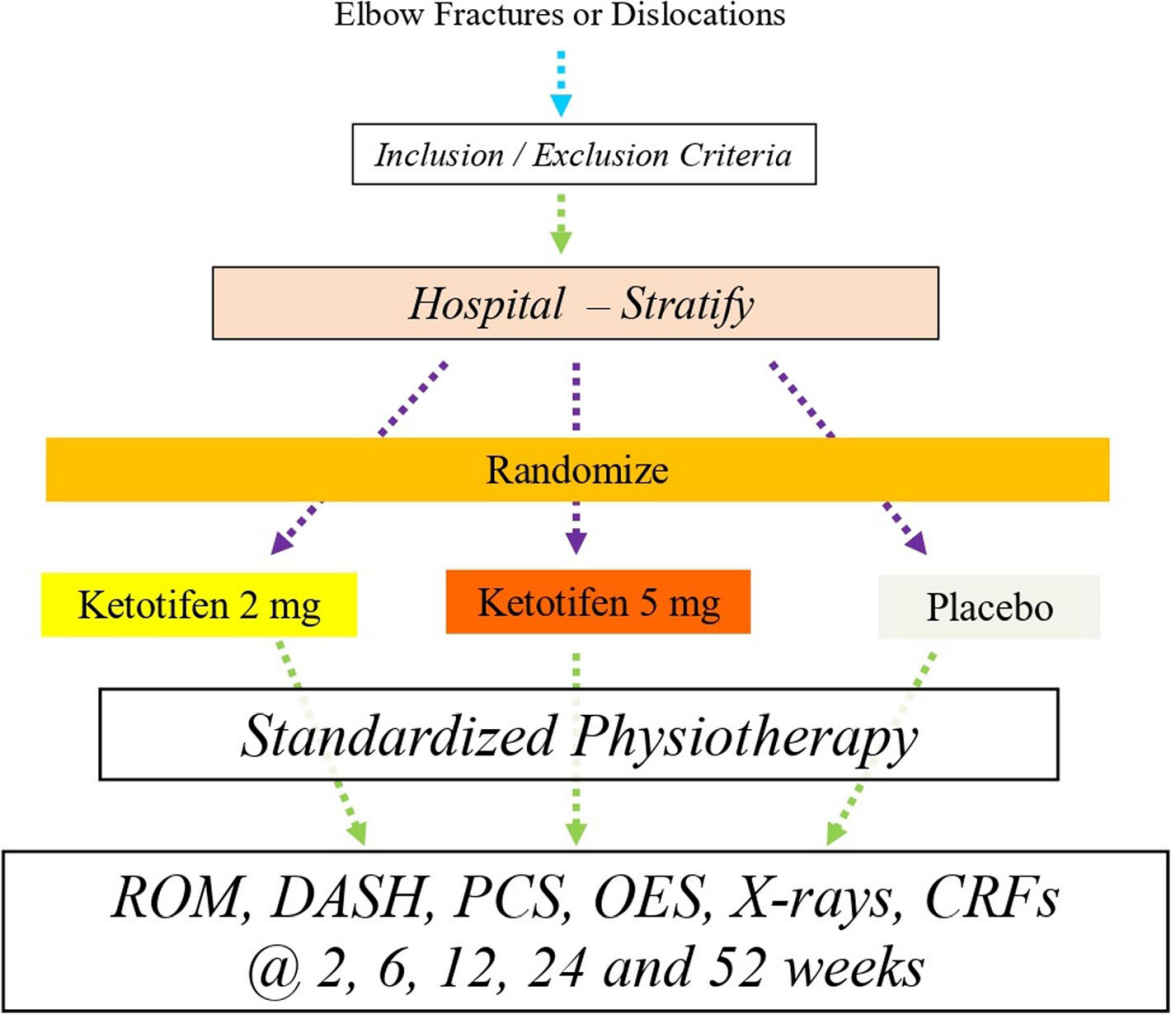
PERK 2 Trial Design

This is a Phase III randomized, controlled blinded multicentre efficacy trial with three parallel groups and a primary endpoint of elbow flexion-extension range of motion (ROM) arc at 12 weeks post-randomization (Fig. 1). Eligible participants will be randomized to receive KF 2 mg, KF 5 mg, or lactose placebo (PL) in a 1:1:1 allocation ratio. The medication is administered orally twice daily for 6 weeks.

## Methods/design

### Participants, interventions, and outcomes

Seventeen academic medical centres from Canada (fourteen) and the United States (three) will be participating in the trial (Appendix 1). The study population of interest are adults (defined as 18 years of age or older) with any combination of distal humerus, or proximal radius or ulna fractures, or elbow dislocations without pre-existing elbow arthritis or contractures (Table 1). A 7 day window from injury to starting medication is based on research indicating that the intervention is most effective if administered early in the healing process [15]. The exclusion criteria are broadly divided in those that predispose to elbow contracture (elbow specific), those that are consequences of using KF or lactose placebo (medication contraindications), and those that interfere with the ability follow the protocol (participant specific (Table 1)). Further, there is a lack of safety evidence for the use of KF during pregnancy or breast feeding.

**Table 1:** Eligibility Criteria

Inclusion Criteria	Exclusion Criteria
• Adults (≥ 18 years or older); skeletally mature • Injury to the elbow with any of the following: o Distal 1/3 humerus fracture (AO/OTA Type 13) o Proximal 1/3 ulna fracture (AO/OTA Type 2 U1) o Proximal 1/3 radius fracture (AO/OTA Type 2R1) o Elbow dislocation • Open fractures with/without nerve injury *can* be included • Participant presents within 7 days or less between injury and trial recruitment • Participant requires operative treatment of the fracture • Participant has a negative urine pregnancy test	• Elbow specific o Pre-existing elbow contracture**,** osteoarthritis, inflammatory arthritis, gout or non-specific monoarticular arthritis of the injured elbow o Inability to mobilize elbow injury within 21 days of injury or surgery o Total Elbow for fracture treatment o Prior Elbow injury or surgery o Bilateral elbow fractures and / or dislocations • Medication contraindications o Oral hypoglycemic medications o History of epilepsy o Lactose intolerance o Any female who is pregnant or nursing o Severe hepatic impairment o Severe renal impairment o Male or female of reproductive age unwilling to use 2 methods of contraception • Participant specific o Unable to maintain follow-up (no fixed address, plans to move out of town in the next year, states unable to comply with protocol, etc.…) o Has cognitive impairment or language difficulties that would impede the reliable completion of questionnaires o Concomitant musculoskeletal or visceral injuries preventing elbow physiotherapy o Unwilling or Unable to provide written informed consent.

### Interventions

Eligible participants will be randomized in equal proportions to receiving KF 2 mg, KF 5 mg, or a PL. This trial follows from the feasibility PERK 1 RCT (Clinicaltrials.gov, NCT01902017) where a dose of 5 mg was used, which is larger than the recommended dose of Ketotifen for the treatment of chronic asthma (1–2 mg twice daily) [11]. In choosing the second dose, the higher end of the recommended dose (2 mg) was selected balancing the effectiveness to prevent contractures and decreasing the chance for side effects (sedation). The medications will be administered orally twice daily for a total of 6 weeks post-randomization. Ketotifen Fumarate is manufactured in 1-mg capsules by TEVA Canada. Bay Area Research Logistics (BARL, Hamilton, Ontario, Canada) specializes in clinical trial medication packaging and distribution and will ensure blinding is achieved by over-encapsulation of each treatment, such that KF and PL capsules will appear identical. Ketotifen Fumarate is an antihistamine and sedation has been reported in 14% of people taking it in post-marketing surveillance [16]. Modifications to the allocated treatment will include discontinuing the capsules for serious suspected adverse reactions such as excessive drowsiness or skin rashes. Adherence to taking the medications will be stressed at the time of dispensing the capsules and reinforced through weekly calls by research personnel, as well as daily diarizing by participant. Adherence assessments with pill counts will be completed at the 2- and 6-week follow-up visits. A relatively short six-week medication administration will facilitate adherence. The only restricted medication that may not be taken simultaneously with KF is antihistamines. If these are required in the first 6 weeks after randomization, the trial medication will be discontinued. There are no rescue medications for KF.

### Assignment of interventions

Screening, randomization and enrollment is organized through the Epidemiology Coordinating and Research Centre (EPICORE) at the University of Alberta. A stratified block randomization scheme by hospital site will be used to assign eligible participants at baseline to KF 2 mg, KF 5 mg, or PL in a 1:1:1 ratio using a computer-generated randomization scheme (i.e. conducted on REDCap over the internet via a desktop computer or a web-enabled smart phone) in randomly assigned block sizes of 3, 6 or 9. All participants, care providers, research personnel, investigators, outcome assessors and data entry personnel will be blinded to intervention groups. Emergency unblinding will be permitted.

### Outcomes

The primary outcome is the range of motion (ROM) in the flexion-extension arc and forearm pronation-supination arc obtained with a hand-held goniometer. Loss of ROM is a major reason for patients to consult physicians following elbow injury. Secondary outcomes include patient reported outcomes measures (PROMs) such as the Disability of the Arm, Shoulder, and Hand questionnaire (DASH), Pain Catastrophizing Scale (PCS) and Oxford Elbow Score (OES). The upper extremity scoring scale DASH is a validated 30-item PROM tool for disorders of the elbow with a range from 0 (least disability) to 100 (most disability). The PCS is a 13-item PROM with aggregates into three subscale scores assessing rumination, magnification, and helplessness that are combined into an overall score that range between 0 (no pain) and 42 (catastrophizing pain) [17–20]. PCS has been used in the assessment of injuries to the elbow, wrist, and hand where results were dependent on these behaviors. The OES is a 12-item valid measure of the outcome of elbow surgery in English and Dutch languages [21, 22]. There are three domains which include elbow function, pain, and social-psychological with values ranging from 0 (greatest severity) to 100 (least severity). The OES has been used in trauma populations [23–26]. It was reported that the OES performed well in assessing elbow surgical outcomes while the DASH was not responsive enough to warrant its exclusive use as an outcome of elbow surgery [27]. The other secondary outcomes include safety (adverse events (AE) and serious adverse events (SAE)), re-operations and fracture healing. For AE, SAE and re-operation, we are interested in the description and number of occurrence, while for fracture healing, our interest is the number of participants with disappearance of radiographic fracture lines overtime.

### Data collection

Potential participants will be identified when scheduled for an operation for their injured elbow through the emergency department, hospital admission, or at an outpatient clinic. The surgeons will be involved in introducing the participants to the trial, discuss the potential concern regarding elbow contracture and the importance of preventing this disabling complication. Surgeons will also obtain permission from the participant for research personnel to contact the participant. Eligible participants who sign informed consent will be allocated to one of the three treatment arms and begin taking the medication the same day (Table 2). Participant assessments include standardized phone calls at 1, 3, 4 and 5 weeks post-randomization to review medication use, ask about adverse events and confirm clinic appointments. Clinic visits, where ROM, PROMs, X-rays, and survey for adverse events are completed, occur at 2, 6, 12, 24 and 52 weeks post-randomization. X-rays will be gathered and stored at the Calgary Image Processing and Analysis Centre (4 views, including obliques). The presence or absence of visible fracture line to indicate healing and location of heterotopic ossification will be adjudicated by 3 independent reviewers. Pill counts will be performed at the 2- and 6-week followup visits (Table 2).

**Table 2:**
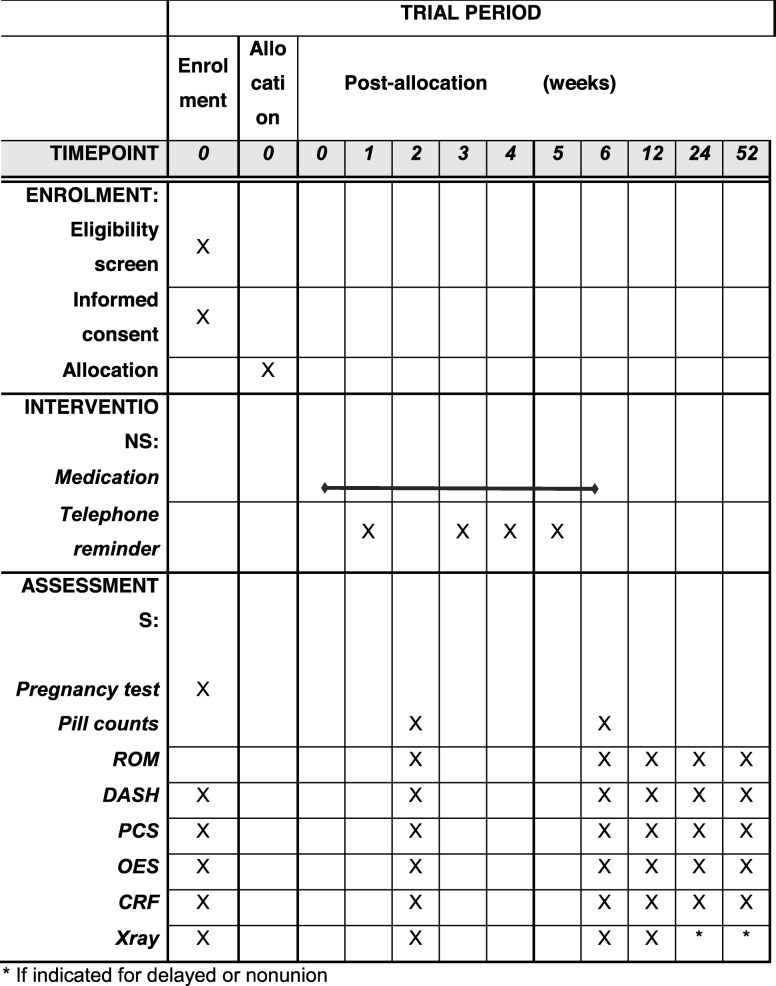
Schedule of enrolment, interventions, and assessments

^a^ If indicated for delayed or nonunion

### Sample size calculation

The sample size was estimated based on analysis of covariance model (ANCOVA) with respect to the mean change from baseline (as represented by the injured elbow at 2-week follow up visits) to flexion-extension arc of motion at week 12, on the basis of a two-sided test at the 0.05 α-level. A total of 702 participants (≈234 participants per arm) is needed to achieve about 95% power to detect a minimum improvement of 10 degrees among the treatment arms, assuming a standard deviation of 19.81, and an overall dropout rate of 11%. This sample size will have about 99.1% statistical power to detect a MCID in mean change (i.e. 10) in DASH scores [28] across treatment groups between baseline and week 12. Furthermore, the total sample size of 702 participants will ensure that we have at least 98% power to detect a minimum of 20% difference in the proportion of participants achieving functional flexion-extension arc of motion between placebo and treatment arms where a functional elbow ROM is defined as at least 30° – 130° flexion-extension arc for activities of daily living [29]. Seventeen centres will provide participants for the trial. The 13 sites outside of Calgary are forecasted to enroll 30 particpants each and the 4 Calgary centres are forecast to enroll the remaining 312 participants (Appendix 1).

### Statistics analysis

Descriptive analysis will be used to summarize ROM measurements, PROMs, safety (AE and SAE), re-operation and radiographic fracture healing. The analysis will follow the intention- to-treat protocol. Analysis of covariance (ANCOVA) will be used to model the impact of KF on ROM (change in 2 and 12 weeks of the injured elbow) adjusting for sex, age, injury classification and site. Similar analysis will be used to assess the impact of KF on in DASH, PCS and OES at 12 weeks, adjusting for these covariates. Mixed-effects regression analysis (effect of time) will be used to examine the impact of KF on changes in ROM and PROMs, adjusting for the earlier covariates. Logistic regression will be used to examine the impact of KF on radiographic fracture line disappearance (i.e. healing) adjusting for sex, age, NSAID, smoking status. Sensitivity analysis will be used to evaluate the robustness of study findings to different methods for handling missing data [30]. Specifically, we will compare study conclusions based on complete data analysis, available data analysis, and use of multiple imputation based on Monte Carlo Markov Chain approach that adjusts for site, age andsex. Exploratory subgroup analyses to confirm treatment effect using different covariates such as range of motion (ROM), sex, age, side of injury is the same as dominant hand, race, concurrent injury and PROM i.e. DASH and OES.

### Data monitoring

The Data Monitoring Committee (DMC), which will be formed, consist of an orthopaedic surgeon, clinical trialist and biostatistician but not involved with the proposed clinical trial in any manner and will be independent of the sponsor. Data will be reviewed twice yearly by the DMC via teleconference including recruitment and follow-up visit rates, SAEs, and descriptive statistics of the baseline demographics. Reports from the DMC will go to the Trial Steering Committee (TSC). The planned interim analysis will be shared with the DMC and a meeting arranged via teleconference for discussion and recommendations with the TSC. An interim analysis will be conducted when 50% of the participants have completed the primary outcome measure (ROM at the week 12 assessment). The DMC will evaluate whether there is an increased risk of KF use compared with PL. The parameters determined a priori to discontinue the trial include:

1. Greater than two times fatal or life-threatening SAEs that are Definitely or Probably related to KF (use Suspected Unexpected Serious Adverse Reaction SUSAR).
2. Greater than three times difference in the DASH and OES between groups.
3. Greater than three times the number of participants with non-functional ROM (30 degrees to 130 degrees flexion-extension arc) between groups.

If any one of the above criteria are met, the DMC will recommend to the TSC that the trial will be terminated prior to completion. The Trial Steering Committee (TSC) will include the Principal Investigator, Trial Biostastician, Project Manager, Orthopaedic Clinical Trialist, and three site Prinicipal Investigators or Project Managers. The TSC will meet quarterly to review trial performance and adjust the RCT as required based on safety and performance inputs. All outcome measures will be evaluated at the completion of the trial when the last participant has reached 52 weeks post-randomization.

### Participant care

Concomitant care covers operations, medications, and rehabilitation/splints. Together, the participant and surgeon will determine the type of operation. This management will not be randomly assigned, but determined by injury characteristics. The operation can be performed before or after randomization; however randomization and first dose administration must occur within 7 days of injury. Analgesia may be provided by acetaminophen, opioids and/ or NSAID as required. Antihistamines use will be excluded for the 6 weeks that participants are taking trial medication since KF is an antihistamine. If possible, a local physiotherapist will direct a standardized home therapy program for all participants consisting of active ROM exercises performed three times per day with 20 repetitions for elbow flexion-extension arc and forearm pronation-supination arc. Progress will be monitored and adherence to home therapy will be reinforced every 2 weeks during the clinic visitation to the local physiotherapist. Stretching splints are instituted once it is clear that physiotherapy alone is insufficient, which occurs a minimum of 12 weeks post-randomization.

### Safety

For the purposes of this trial, AEs will be defined as any unfavourable medical occurrence that a participant experiences, including sign, symptom or disease that occurs during participation in the trial whether or not considered trial medication related [7, 8, 28, 31–36]. Serious Adverse Events will be defined as any untoward medical occurrence that: results in death or is life-threatening; requires inpatient hospitalization or prolongation of existing hospitalization; leads to persistent or significant disability and/or incapacity; causes a congenital anomaly and/or birth defect. All AEs and SAEs will be collected as a secondary outcome of the trial in a standardized format on the case report forms (CRFs). Suspected Adverse Reaction (SAR) is a type of AE where there is a reasonable possibility that the trial medication KF caused the AE. For all AEs, a temporal relationship will be observed in relation to the KF administration. Ketotifen Fumarate is administered over 6 weeks, and a 1-week washout period will be included. Thus, to be considered a SAR, the AE would have to occur within 7 weeks of randomization. The “definitely related” and “probably related” designations would indicate the AE is a SAR. For pregnancy, any conception that arises within the 6 weeks and/or within a 3-month interval after the 6-week course of KF will be followed to ascertain whether any birth defects, congenital anomalies or loss of pregnancy occurred. Unblinding will occur in order to ascertain what the participant was randomized in order to provide counselling on what the fetus may have been exposed to KF or PL [31–35, 37–40]. All AEs or SAEs will be classified as expected or unexpected. Expected AEs are defined in the product monograph (PM) for KF and include recognized complications of an elbow fracture population undergoing an operation for the injury [36]. Unexpected AEs or SAEs are all other AEs or SAEs not defined as expected. Suspected unexpected serious adverse reactions (SUSAR) are defined as unexpected SAEs where there is causal relation between the SAE and use of KF.

## Discussion

Currently, if contractures develop then only solution is an operation followed by 6–9 months of rehabilitation. An oral medication used during the recovery from the original injury to prevent post-traumatic contracutres is a simpler and safer approach. Contractures complicate other orthopaedic conditions or procedures and positive results in post-trauamtic contractures in elbow may warrant study of the use of KF in preventing contractures in other orthopaedic conditions or procedures. Therefore, we believed that the PrEvention of Posttraumatic Joint contractuRes With Ketotifen 2 (PERK 2) trial will provide evidence for a treatment (Ketotifen) to prevent contractures following joint injuries. If an improvement in ROM is observed, the treatment will have a significant impact on quality of life. A multi-centre design involving seventeen sites is planned to achieve a recruitment rate of 234 participants per year over 3 years to obtain a total of 702 participants. The multi-dosage regimen will provide an opportunity to determine the optimal dosage for treatment. The safety of KF for a new indication will be assessed, including radiographic fracture healing.

## Supplementary information

**Additional file 1.** Trial Sites.

## Data Availability

All data generated or analysed during this study are included in this published article [and its supplementary information files].
