# Supplementary material for: PrEvention of posttraumatic contractuRes with Ketotifen 2 (PERK 2) – protocol for a multicenter randomized clinical trial
Source: BMC Musculoskelet Disord. 2020 Feb 24;21:123. doi: 10.1186/s12891-020-3139-2 (PMC7041204; doi:10.1186/s12891-020-3139-2)
Supplement: Supplementary file 1 — Additional file 1. Trial Sites. [file 12891_2020_3139_MOESM1_ESM.docx]

**Appendix**

1. **Trial Sites**

Peter Lougheed Centre, Calgary, AB

3500 26 Avenue NE

Calgary, AB T1Y 6J4

Canada

403-943-5556

Foothills Medical Centre, Calgary, AB

1403 29 Street NW

Calgary, AB T2N 2T9

Canada

403-944-2932

South Health Campus, Calgary, AB

4448 Front Street SE

Calgary, AB T3M 1M4

Canada

403-956-3687

Rockyview General Hospital, Calgary, AB

7007 14 Street SW

Calgary, AB T2V 1P9

Canada

403-536-3725

Sturgeon Community Hospital, St. Albert, AB

201 Boudreau Road

St. Albert, AB T8N 6C4

Canada

780-492-2398

University of Vermont Medical Centre, Burlington, VT

111 Colchester Avenue

Burlington, VT 05401

USA

802-656-8396

Carolinas Medical Centre, Charlotte, NC

1000 Blythe Boulevard

Charlotte, NC 28203-5812

USA

704-355-6969

McGill University Health Centre, Montreal, QC

Montreal General Hospital

1650 Cedar Avenue

Montreal, QC H3G 1A4

Canada

514-934-1934 ext. 43386

St. Joseph’s Hospital, London, ON

339 Windermere Road

London, ON N64 5A5

Canada

519-646-6100 ext. 64640

St. Michael’s Hospital, Toronto, ON

30 Bond Street

Toronto, ON M5B 1W8

Canada

416-864-6060 ext. 2608

Sunnybrook Health Sciences Centre, Toronto, ON

2075 Bayview Avenue

Toronto, ON M4N 3M5

Canada

416-505-7686

University of Maryland Medical Centre, Baltimore, MD

22 S Greene Street

Baltimore, MD 21201

USA

704-355-6969

The Ottawa Hospital - Civic Campus, Ottawa, ON

1053 Carling Avenue

Ottawa, ON K1Y 4E9

Canada

613-737-8899 ext. 19217

The Ottawa Hospital – General Campus, Ottawa, ON

501 Smyth Road

Ottawa, ON K1H 8L6

Canada

613-737-8785 ext. 79839

QEII Health Science Centre, Halifax, NS

1799 Robie Street

Halifax, NS B3H 3G1

Canada

902-473-3161

St. Paul’s Hospital, Vancouver, BC

1081 Burrard Street

Vancouver, BC V6Z 1Y6

Canada

- - 1. ext. 66550

Royal Columbian Hospital, New Westminster, BC

330 East Columbia Street

New Westminster, BC V3L 3W7

Canada

604-520-4855
